# Smartphone CBT engagement and depressive symptoms: secondary analysis of the RESiLIENT trial using a time-varying exposure approach

**DOI:** 10.1017/S0033291726104279

**Published:** 2026-05-13

**Authors:** Yan Luo, Kosuke Inoue, Aran Tajika, Rie Toyomoto, Masatsugu Sakata, Tatsuo Akechi, Masaru Horikoshi, Hisashi Noma, Toshi A. Furukawa

**Affiliations:** 1 Department of Next-Generation Organ Transplantation, The University of Tokyo Graduate School of Medicine Faculty of Medicine, Japan; 2 Center for Medical Education and Internationalization, Kyoto University Graduate School of Medicine Faculty of Medicine, Japan; 3 Department of Health Promotion and Behavioral Sciences, Kyoto University Graduate School of Medicine Faculty of Medicine, Japan; 4 Department of Neurodevelopmental Medicine, Nagoya City University Graduate School of Medical Sciences and Medical School, Japan; 5 International Institute for Integrative Sleep Medicine (WPI-IIIS), Tsukuba Institute for Advanced Research (TIAR), University of Tsukuba, Japan; 6 Department of Psychiatry and Cognitive-Behavioral Medicine, Nagoya City University Graduate School of Medical Sciences and Medical School, Japan; 7 Department of Human Sciences, Musashino University, Japan; 8 Department of Interdisciplinary Statistical Mathematics, Institute of Statistical Mathematics, Japan; 9 Office of Institutional Advancement and Communications, Kyoto University, Japan

**Keywords:** behavioral activation, causal inference, cognitive behavioral therapy, digital mental health, engagement, subthreshold depression, time-varying exposure

## Abstract

**Background:**

Smartphone-based cognitive behavioral therapy (CBT) programs offer accessible interventions for subthreshold depression, yet engagement needed for meaningful benefit remains unclear. We examined how lesson and worksheet engagement relate to depressive symptom improvements in a behavioral activation (BA) intervention, accounting for time-varying confounders.

**Methods:**

This secondary analysis included 298 adults assigned to the BA arm of the RESiLIENT trial, a randomized controlled trial in Japan. Lesson and worksheet completion were treated as time-varying exposures, each yielding four engagement patterns: *minimal (Few-Few)*, *early (Many-Few)*, *late (Few-Many)*, and *consistently high (Many-Many).* Outcomes were depressive symptom changes measured by the Patient Health Questionnaire-9 (PHQ-9) at weeks 6 and 26. We applied the parametric g-formula to estimate counterfactual PHQ-9 changes under each pattern, adjusting for baseline and time-varying confounders.

**Results:**

Early lesson engagement during weeks 0–3 was associated with larger PHQ-9 reductions at both weeks 6 and 26, even when later engagement declined (*Many-Few* vs. *Few-Few*: week 6: −1.47 [95% CI −2.52 to −0.53]; week 26: −1.27 [−2.53 to −0.17]). In contrast, higher worksheet engagement was linked to improved PHQ-9 at week 6, with maximal benefit among consistently high engagers (*Many-Many* vs. *Few-Few*: −1.25 [−2.17 to −0.44]) and late engagers (*Few-Many* vs. *Few-Few*: −1.18 [−2.20 to −0.08]), but not persist to week 26.

**Conclusions:**

Greater engagement with smartphone-delivered BA is associated with larger symptom reductions. Early lesson engagement drives sustained benefit, whereas worksheet engagement did not persist. These findings may guide digital CBT design by emphasizing early lesson completion alongside concurrent skill practice.

## Introduction

Cognitive behavioral therapy (CBT) is a well-established intervention for depressive symptoms (Cuijpers et al., 2014a; Hofmann et al., 2012) and has demonstrated benefits for individuals with subthreshold depression (Cuijpers et al., 2014b; Harrer et al., 2025). However, low treatment uptake for subthreshold depression (Lee et al., 2019) has prompted interest in more accessible formats, including internet- and smartphone-based CBT programs (Andersson et al., 2019). These programs typically deliver structured modules or lessons that mirror face-to-face CBT and often include homework assignments similar to conventional therapy. In fact, accumulating evidence now supports the efficacy of digital CBT for subthreshold depression (Karyotaki et al., 2021; Zhou et al., 2016).

Despite demonstrated efficacy, the optimal level of engagement required to achieve meaningful benefit remains unclear. Engagement, in this context, refers to participants’ uptake and adherence to the program, including completing lessons and practicing skills through homework (Torous et al., 2018). Unlike face-to-face CBT, smartphone-based programs lack direct therapist interaction, which may result in low engagement and diminished treatment effects (Gan et al., 2021; Torous et al., 2018). Although higher utilization is often assumed to produce better outcomes (*the more use, the better*), previous studies have reported mixed findings: some observed that greater engagement predicts better outcomes, whereas others found no consistent association between engagement and clinical improvements (Boucher & Raiker, 2024; Donkin et al., 2011; Sieverink, Kelders, & van Gemert-Pijnen, 2017).

While differences in engagement measures and study context may partly explain these conflicting findings, methodological limitations are also likely to play a substantial role. First, the relationship between engagement and depressive symptoms may be nonlinear, meaning that either very low or very high levels of engagement might not yield optimal benefit (Boucher & Raiker, 2024). Yet, many prior studies have reported only simple correlation coefficients under the assumption of linearity (Donkin et al., 2011). Second, even in randomized controlled trials (RCTs) of smartphone-based CBT, engagement itself is usually not randomized. Thus, analyses of the association between engagement and clinical outcomes are observational in nature. Most previous studies have adjusted only for baseline confounders and have not accounted for time-varying confounding (Elkes et al., 2024). For instance, participants may increase program use either due to persistent symptoms, which prompt more engagement, or due to rapid improvement, which enhances motivation to engage (Hamitouche et al., 2025). In this case, treatment response over time acts as a time-varying confounder, and ignoring such dynamics leads to biased estimates for the causal relationship between engagement and outcomes (Hernán & Robins, 2020).

To address these issues, we applied a time-varying exposure approach to examine the relationship between engagement in a smartphone CBT app and depressive symptoms, using data from the RESiLIENT trial, a large-scale RCT of subthreshold depression in the general Japanese population (Furukawa et al., 2025b). While the trial included multiple CBT skills in different arms, the present study focused on the behavioral activation (BA) skill, which has been shown to alleviate depressive symptoms in subthreshold depression (Furukawa et al., 2021). Measuring engagement in digital interventions is challenging due to their multidimensional nature, encompassing module adherence, access time, and self-reported practice (Lipschitz et al., 2023; Smith et al., 2025). In this study, we used lesson completion and homework (worksheet) completion as two indicators of engagement and examined them separately. They were analyzed as time-varying exposures, allowing adjustment for time-varying confounders such as changes in depressive symptoms, motivation, and life events. This approach provides a more reliable assessment of how engagement over time causes outcomes on average, independent of symptom trajectories or other intervening factors, and may inform future optimization of smartphone-based CBT apps.

## Methods

This study is a secondary analysis of data from the RESiLIENT trial (Resilience Enhancement with Smartphone in Living ENvironmeTs) (Furukawa et al., 2025b). The original RCT was approved by the Ethics Committee of the Kyoto University Graduate School and Faculty of Medicine (C1556). Details of the trial protocol (Furukawa et al., 2023) and the primary results (Furukawa et al., 2025b) have been published elsewhere. This report follows the STROBE guideline for reporting observational studies (von Elm et al., 2007).

### Study design

Although data from an RCT were used, this analysis focused on a single intervention arm only and thus employed an observational cohort design.

### Setting

Participants in the RESiLIENT trial were recruited across Japan between 5 September 2022 and 21 February 2024 through health insurance societies, companies, local governments, and online advertisements. Participants were randomized to various intervention arms that delivered certain CBT skills via a smartphone application. They were asked to complete the Patient Health Questionnaire-9 (PHQ-9) (Kroenke, Spitzer, & Williams, 2001) a self-reported scale of depression severity, weekly until week 6, and then every 4 weeks until week 50.

### Participants

Eligible participants for this secondary analysis were adults aged 18 years or older with a PHQ-9 ≥ 5 at screening and randomization (week 0); among those with a PHQ-9 ≥ 10, participants scoring 2 or 3 on item 9 (suicidality) were excluded. Individuals receiving treatment from mental health professionals at screening were excluded. The trial included nine active arms and three control arms, but this study focuses on participants who were randomized to the BA arm. We selected BA because lesson schedules and worksheet content varied across interventions, whereas BA is a well-established CBT component with coherent lesson content and straightforward homework tasks, making the results also easier to interpret.

### Exposures

BA skills were delivered through lessons explaining the skills and worksheets for practicing them. Therefore, completion of these two components was the two exposures we investigated in this study:

1. *Lesson completion:* Participants were expected to complete seven lessons in total. The first lesson, a nonspecific introduction, was typically completed on the day of randomization during the informed consent session. The remaining six lessons explained the BA skill that encourages engagement in pleasurable activities in line with the ‘outside-in’ principle, with one lesson ideally completed each week. The second through fifth lessons covered troubleshooting common pitfalls, provided tips for enjoying BA, and discussed how to use BA in daily lives. The sixth and final lesson served as a review of all skills covered.
2. *Worksheet completion:* Participants practiced BA skills as homework by completing worksheets within the app, which involved planning and recording pleasurable activities. Each recorded activity counted as one worksheet. There was no limit to the number of worksheets participants could submit.

Beyond the lessons and worksheets, all participants received weekly encouragement emails, which followed a standard template but were tailored by trial coordinators according to each participant’s progress. However, coordinators were explicitly prohibited from providing any therapeutic content.

### Outcomes

The outcomes were changes in depression severity from baseline to week 6 and to week 26, assessed by the PHQ-9 (Kroenke, Spitzer, & Williams, 2001). PHQ-9 is a nine-item self-report questionnaire covering core depressive symptoms experienced over the past week. Total scores range from 0 to 27, with higher scores indicating greater symptom severity.

### Statistical analysis

We first summarized participants’ baseline characteristics. To examine the association between lesson and worksheet completion and depressive symptoms, we used two approaches:

#### Fixed exposure approach

For the fixed exposure approach, we used conventional linear regression models. Lesson completion was treated as a continuous variable, defined as the total number of lessons completed by week 6, given its limited range from 1 to 7 and absence of extreme values, and was examined in relation to PHQ-9 at weeks 6 and 26 under a linear specification. For worksheet completion, as the total numbers of worksheets completed by weeks 6 and 26 showed a wide range with extreme values, they were treated as categorical variables (≤ 10, 11–15, 16–20, 21–30, 31–40, and ≥ 41) and examined in relation to PHQ-9 at weeks 6 and 26, respectively. These models adjusted for baseline confounders selected using a directed acyclic graph (DAG; Supplementary Materials S1.2 (A)), including age, gender, education, employment, number of cohabitants, past or current treatments for mental health, personality traits assessed by the Big-5 questionnaire (Namikawa et al., 2012; Toyomoto et al., 2022; Wada, 1996), baseline PHQ-9, social support (Assessment of Signal Cases [ASC] – Social Support) (Probst et al., 2014), life events (ASC – Life difficulties) (Probst et al., 2014), and motivation for smartphone CBT (ASC – Motivation) (Probst et al., 2014).

#### Time-varying exposure approach

As lesson and worksheet completion were behaviors that could change over time, a conventional regression approach may yield biased estimates for the causal effects of post-baseline exposures because it does not adjust for time-varying confounders that are themselves influenced by prior exposure (Daniel et al., 2013). To address this, we used the parametric g-formula, which appropriately adjusts for time-varying confounders by modeling the joint distribution of exposures, confounders, and outcomes across multiple time points (Hernán & Robins, 2020; Robins, 1986).

We defined time-varying exposures as categorical variables (Supplementary Materials S1.1, Cf. Table 3). For lesson completion, we classified participants into four patterns based on the number of lessons completed between weeks 0–3 (Few: ≤ 3 lessons; Many: ≥ 4 lessons) and weeks 3–6 (Few: ≤ 2 lessons; Many: ≥ 3 lessons): *Few-Few (minimal engagement)*, *Few-Many (late engagement)*, *Many-Few (early engagement)*, and *Many-Many (consistently high engagement).* Thresholds were selected to maximize clinical interpretability while maintaining statistical feasibility. The intervention consisted of seven lessons and was designed to be completed at a rate of approximately one lesson per week, with the first lesson delivered at randomization. Under this design, on-schedule engagement corresponds to completing four lessons by week 3 and the remaining three lessons between weeks 3 and 6; thus, the *Many-Many* group reflects adhering to the intended schedule throughout the 6 weeks. These thresholds also ensured reasonably sufficient numbers of participants in each category.

Worksheet completion was classified similarly for the outcome at week 6, for which four patterns were defined according to the number of worksheets completed in weeks 0–3 (Few: ≤10 worksheets; Many: ≥ 11 worksheets) and weeks 3–6 (Few: ≤ 5 worksheets; Many: ≥ 6 worksheets). For the outcome at week 26, worksheet completion patterns also incorporated the number completed between weeks 6 and 26 (Few: ≤ 3; Many: ≥ 4), resulting in eight trajectory patterns. This differed from lesson completion, for which lessons completed during weeks 6–26 were not included in the analysis because the total number of lessons was fixed, whereas there was no limit to the number of worksheets that could be completed. Worksheet engagement was self-directed and exhibited substantially greater variability. Therefore, we selected thresholds around the 40th percentile of the observed distributions to represent a minimally engaged reference group while maintaining adequate sample sizes across categories.

To implement the g-formula, we specified a series of parametric regression models to estimate the conditional distributions of time-varying exposures, time-varying confounders, and outcomes at each time point, which together characterize the joint data-generating process. Baseline confounders were the same as in the fixed exposure analysis, while time-varying confounders included PHQ-9 score, life difficulties, and motivation for smartphone CBT, measured at week 3 (Supplementary Materials S1.2(B)). The g-formula relies on several key assumptions including (1) consistency – the observed outcome under one pattern equals the counterfactual outcome if the exposure pattern is followed; (2) positivity – each individual has a nonzero probability of receiving every exposure pattern conditional on the covariates; (3) no unmeasured confounding – all potential confounders are measured and correctly modeled; and (4) correct model specification – the parametric models adequately capture the true relationships among variables.

We estimated the expected mean PHQ-9 scores under each hypothetical exposure scenario corresponding to each completion pattern by simulating counterfactual outcomes in which exposure values were deterministically set according to the predefined engagement patterns, while the fitted models for time-varying confounders and outcomes were used to simulate their evolution over time. Simulations were based on 10,000 Monte Carlo samples, and 1000 bootstrap samples were used to compute 95% confidence intervals (CIs) using the percentile method. To examine the relationship of lesson and worksheet completion with PHQ-9, our primary contrasts of interest were the mean differences in PHQ-9 change scores comparing *Few-Many*, *Many-Few*, and *Many-Many* exposure patterns against the *Few-Few* reference regime. These contrasts quantify the depression severity associated with completion patterns relative to minimal exposure and constitute the primary estimands of interest.

##### Additional exploratory analyses

1. In addition to the primary contrasts against the *Few-Few* reference regime, we conducted secondary exploratory contrasts to compare nonreference exposure patterns. Specifically, we compared *Many-Many* versus *Many-Few* to assess the incremental contribution of late-phase engagement, given high early participation, and *Many-Many* versus *Few-Many* to assess the incremental contribution of early engagement, given high late-phase participation. These secondary contrasts were exploratory in nature and not designed to formally establish superiority between nonreference engagement patterns.
2. For the analysis of worksheet completion and PHQ-9 change at week 6, we conducted two sensitivity analyses: (a) to assess robustness to finer categorization beyond the primary dichotomization (40th-percentile cutoffs, which produced a relatively large “Many” group), we further subdivided the “Many” group into “Moderate” and “Many” categories at cutoffs that roughly split the observed values into two comparably sized subgroups, resulting in three categories per period and nine patterns; (b) we defined thresholds using tertiles, also yielding nine patterns (Supplementary Materials S1.1).

Participants with missing data on any covariates or outcomes were excluded from the analysis, as missingness was minimal in the original RESiLIENT trial, with follow-up proportions of 96.8% at week 6 and 90.1% at week 26 (Furukawa et al., 2025a, 2025b). All analyses were conducted in R (version 4.2.3) (R Core Team, 2013), and the package gfoRmula (version 1.1.1) was used for time-varying exposure analyses.

## Result

### Participant characteristics

A total of 327 participants were randomized to the BA arm in the RESiLIENT trial, among whom 298 (91.1%) had all data available for analysis. Table 1 shows the baseline characteristics for these participants.

**Table 1.** Baseline characteristics of participants in the analysis

	Total (*n* = 298)
Age, mean (SD)	45.0 (10.7)
Sex, *n* (%)	
Female	150 (50.3)
Male	146 (49.0)
Other	2 (0.7)
Number of household members (including self), mean (SD)	2.74 (1.30)
Education, university or higher, *n* (%)	209 (70.1)
Employment, employed, *n* (%)	272 (91.3)
Big-5 personality traits, mean (SD) [score range: 1–5]	
Neuroticism	3.61 (0.78)
Extraversion	2.95 (0.93)
Openness	3.17 (0.77)
Conscientiousness	3.01 (0.79)
Agreeableness	3.19 (0.87)
Assessment of signal cases, mean (SD) [score range: 0–3]	
Social support	1.33 (0.74)
Life difficulties	0.93 (0.59)
Motivation	2.21 (0.37)
PHQ-9, mean (SD) [score range: 0–27]	7.91 (2.55)
Past or current treatments for mental health, *n* (%)	98 (32.9)

*Note*: PHQ-9, Patient Health Questionnaire-9; SD, standard deviation.

### Exposure 1: Lesson completion

#### Fixed exposure approach

Figure S1 in Supplementary Materials S2.1 shows the distribution of total lessons completed. By week 6, 92 participants (30.8%) had completed all seven lessons as ideally scheduled. By week 26, this number increased to 211 (70.8%). After adjusting for baseline confounders, each additional lesson completed by week 6 was associated with a 0.28-point greater reduction in PHQ-9 score at week 6 (95%CI: −0.49 to −0.06, *p* = 0.01). This trend remained at week 26, although the 95% CI included the null (−0.23 [−0.51 to 0.06, *p* = 0.11]).

#### Time-varying exposure approach

Table S1 in Supplementary Materials S2.1 presents the baseline characteristics of participants for the four patterns based on lesson completion treated as a time-varying exposure.

**Figure 1.**
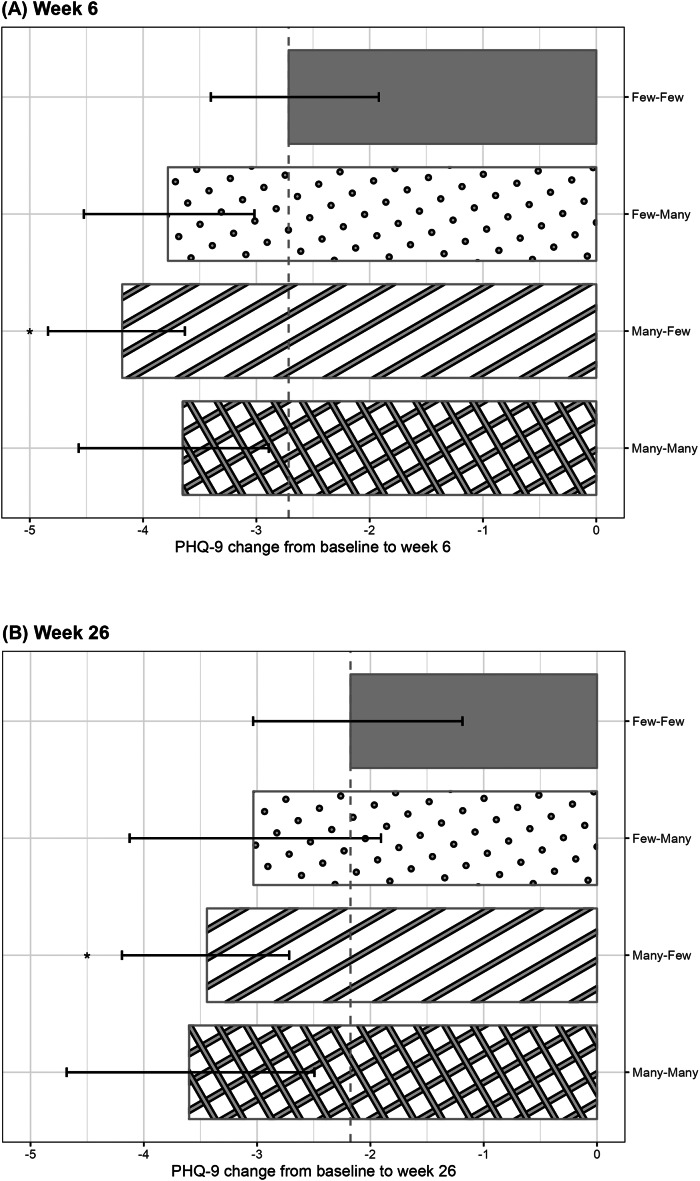
Association between lesson completion patterns and changes in PHQ-9 scores. (a) Week 6. (b) Week 26. The bars marked with * indicate that the bootstrap 95% confidence intervals for the mean difference between those groups and the *Few–Few* pattern do not include the null value, suggesting greater improvements compared with the minimal engagement group. *Abbreviations:* PHQ-9, Patient Health Questionnaire-9.

Figure 1 and Table 2 show the estimated changes in PHQ-9 scores from baseline for each lesson completion pattern, adjusted for both baseline and time-varying confounders. Compared to the *Few-Few* pattern, all other groups showed larger mean reductions in PHQ-9 scores. For the *Many-Few* versus *Few-Few* comparison, representing the contribution of early engagement in the context of low later participation, the estimated mean differences indicated greater improvements at both week 6 (−1.47 [−2.52 to −0.53]) and week 26 (−1.27 [−2.53 to −0.17]). For the *Few-Many* versus *Few-Few* comparison, representing the additional contribution of late engagement following low early participation, the estimates suggested improvements, though 95% CI included the null (week 6: −1.07 [−2.20 to 0.06]; week 26: −0.86 [−2.35 to 0.48]). In exploratory secondary contrasts, no clear evidence of incremental contribution from later participation among those with high early engagement was found (*Many-Many* vs. *Many-Few*: week 6, 0.53 [−0.48 to 1.39]; week 26, −0.16 [−1.36 to 1.03]). Meanwhile, when later participation was high, no evidence of additional contribution of early participation was observed at week 6 (*Many-Many* vs. *Few-Many*: 0.13 [−0.99 to 1.17]), and only a small, imprecisely estimated difference was observed at week 26 (−0.57 [−2.14 to 0.84]).

**Table 2.** Results for time-varying lesson completion

Pattern	*N*	Number of lessons in weeks 0–3	Number of lessons in weeks 3–6	Total number of lessons by week 6, median (Q1, Q3)	Week 6		Week 26	
					Estimated PHQ-9 change from baseline	Estimated mean difference	Estimated PHQ-9 change from baseline	Estimated mean difference
*Few-Few* [Minimal]	85	≤ 3	≤ 2	4 (3, 5)	−2.72 (−3.40 to −1.92)	Reference	−2.18 (−3.04 to −1.19)	Reference
*Few-Many* [Late]	61	≤ 3	≥ 3	6 (5, 6)	−3.78 (−4.52 to −3.02)	−1.07 (−2.20 to 0.06)	−3.03 (−4.12 to −1.91)	−0.86 (−2.35 to 0.48)
*Many-Few* [Early]	85	≥ 4	≤ 2	6 (5, 6)	−4.18 (−4.84 to −3.63)	−1.47 (−2.52 to −0.53)*	−3.44 (−4.19 to −2.72)	−1.27 (−2.53 to −0.17)*
*Many-Many* [High]	67	≥ 4	≥ 3	7 (7, 7)	−3.65 (−4.57 to −2.89)	−0.93 (−2.17 to 0.11)	−3.60 (−4.68 to −2.49)	−1.42 (−3.02 to 0.04)

*Note*: PHQ-9, Patient Health Questionnaire-9. * indicate that the bootstrap 95% confidence intervals for the mean difference between those groups and the Few–Few pattern do not include the null value, suggesting greater improvements compared with the minimal engagement group.

### Exposure 2: Worksheet completion

#### Fixed exposure approach

Figure S2 in Supplementary Materials S2.1 shows the distribution of total worksheets completed. By week 6, participants had completed between 0 and 505 worksheets, with a median of 19. By week 26, the total ranged from 0 to 982, with a median of 24. Table S2 in Supplementary Materials S2.2 summarizes the results of the linear regression examining the association between the total number of completed worksheets and changes in PHQ-9 scores. After adjusting for baseline confounders, a higher number of worksheets was generally associated with greater PHQ-9 reductions at week 6 (Table S2). However, the association was not dose–response at week 26, with a total number of 31–40 worksheets associated with the largest improvements.

#### Time-varying exposure approach

Table S3 in Supplementary Materials S2.2 presents baseline characteristics for the four predefined worksheet completion patterns over time.

**Figure 2.**
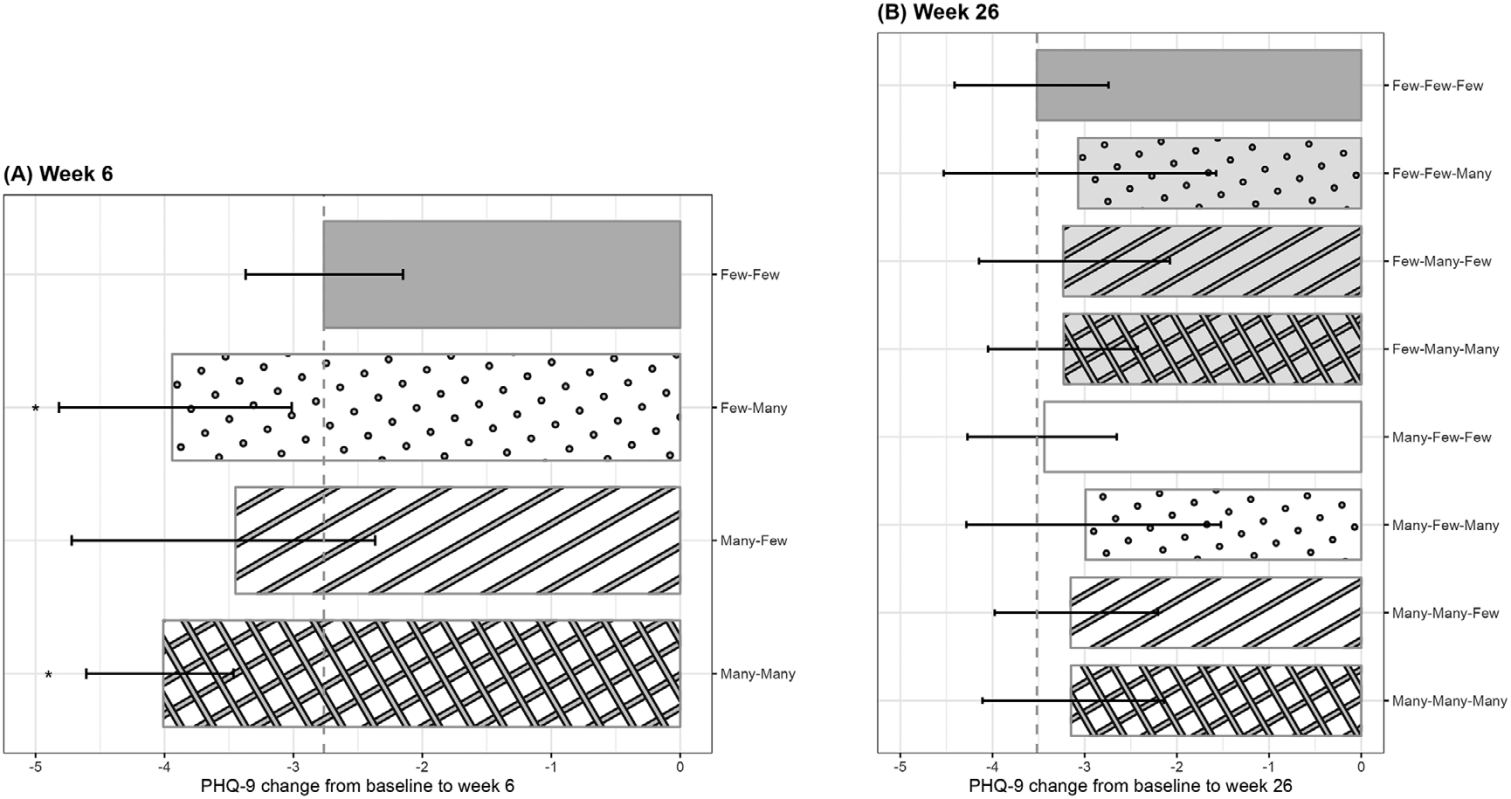
Association between worksheet completion patterns and changes in PHQ-9 scores. (a) Week 6. (b) Week 26. The bars marked with * indicate that the bootstrap 95% confidence intervals for the mean difference between those groups and the *Few–Few* pattern do not include the null value, suggesting greater improvements compared with the minimal engagement group. *Abbreviations:* PHQ-9, Patient Health Questionnaire-9.

Figure 2, Table 3, Figure S3 and Table S4 in Supplementary Materials S2.2 present the estimated changes in PHQ-9 scores from baseline for each pattern, adjusted for both baseline and time-varying confounders. At week 6, relative to the *Few-Few* pattern, all three other patterns demonstrated greater PHQ-9 reductions, with the largest estimate observed for the *Many-Many* pattern (−1.25 [−2.17 to −0.44]). Among participants with minimal worksheet completion during the first 3 weeks, greater later engagement was associated with larger reductions (*Few-Many* vs. *Few-Few*: −1.18 [−2.20 to −0.08]). In exploratory secondary contrasts, the incremental contribution of later engagement among those with high early participation was estimated as −0.56 [−1.79 to 0.77] (*Many-Many* vs. *Many-Few*). When later engagement was high, no additional contribution of early participation was observed (*Many-Many* vs. *Few-Many*: −0.07 [−1.12 to 0.93]). Sensitivity analyses using finer categorizations of nine patterns also showed that greater worksheet engagement during the second half of the intervention was associated with larger reductions in PHQ-9 at week 6 (Figure S3 and Table S4 in Supplementary Materials S2.2). This pattern was observed both when the primary cutoffs were refined (1) by subdividing the “Many” category, and (2) when engagement was defined using tertiles. In the tertile-based analysis, in addition to *Moderate–Moderate*, *Moderate-Many*, *Many-Moderate*, and *Many-Many*, *Few-Moderate* and *Few-Many* also showed lower PHQ-9 scores compared to the *Few-Few* pattern.

**Table 3.** Results for time-varying worksheet completion

Pattern	*N*	Number of worksheets in weeks 0–3	Number of worksheets in weeks 3–6	Number of worksheets in weeks 6–26	Total number of worksheets, median (Q1, Q3)	Estimated PHQ-9 change from baseline	Estimated mean difference
Week 6							
*Few-Few* [Minimal]	75	≤ 10	≤ 5	-	9 (6.5, 11)	−2.76 (−3.37 to −2.15)	Reference
*Few-Many* [Late]	55	≤ 10	≥ 6	-	16 (13, 18)	−3.94 (−4.82 to −3.01)	−1.18 (−2.20 to −0.08)*
*Many-Few* [Early]	40	≥ 11	≤ 5	-	17 (15.75, 20.25)	−3.45 (−4.72 to −2.37)	−0.69 (−2.02 to 0.53)
*Many-Many* [High]	128	≥ 11	≥ 6	-	36 (26, 59.5)	−4.01 (−4.61 to −3.47)	−1.25 (−2.17 to −0.44)*
Week 26							
*Few-Few-Few*	59	≤ 10	≤ 5	≤ 3	10 (8, 12)	−3.52 (−4.41 to −2.74)	Reference
*Few-Few-Many*	16	≤ 10	≤ 5	≥ 4	15.5 (12.75, 18.25)	−3.07 (−4.53 to −1.57)	0.45 (−1.10 to 2.07)
*Few-Many-Few*	30	≤ 10	≥ 6	≤ 3	16.5 (12.25, 18)	−3.23 (−4.15 to −2.08)	0.29 (−0.93 to 1.55)
*Few-Many-Many*	25	≤ 10	≥ 6	≥ 4	27 (19, 33)	−3.23 (−4.05 to −2.42)	0.29 (−0.74 to 1.41)
*Many-Few-Few*	26	≥ 11	≤ 5	≤ 3	18 (16, 19.75)	−3.44 (−4.27 to −2.65)	0.08 (−0.27 to 0.42)
*Many-Few-Many*	14	≥ 11	≤ 5	≥ 4	29 (26.25, 39.25)	−2.99 (−4.28 to −1.52)	0.53 (−1.03 to 2.22)
*Many-Many-Few*	51	≥ 11	≥ 6	≤ 3	34 (26, 46.5)	−3.15 (−3.98 to −2.21)	0.37 (−0.79 to 1.55)
*Many-Many-Many*	77	≥ 11	≥ 6	≥ 4	70 (42, 134)	−3.15 (−4.11 to −2.14)	0.37 (−0.83 to 1.65)

*Note:* PHQ-9, Patient Health Questionnaire-9. * indicate that the bootstrap 95% confidence intervals for the mean difference between those groups and the Few–Few pattern do not include the null value, suggesting greater improvements compared with the minimal engagement group.

At week 26, compared to the minimal worksheet completion pattern, there was no clear evidence of sustained differences in depressive symptoms across worksheet completion patterns.

## Discussion

In this secondary analysis of 298 participants with subthreshold depression randomized to the BA skills arm of an RCT of a smartphone-based CBT app, we applied a time-varying exposure approach to examine how lesson and worksheet completion related to improvements in depressive symptoms, while accounting for time-varying confounders. For lesson completion, greater engagement was associated with sustained reduction in PHQ-9, with the strongest evidence observed among participants who engaged intensively early, even if engagement later declined (*Many-Few* vs. *Few-Few*: week 6, −1.47 [−2.52 to −0.53]; week 26, −1.27 [−2.53 to −0.17]). For worksheet completion, higher engagement was linked to greater short-term PHQ-9 improvements at week 6. The largest reductions were observed among participants with consistently high participation (*Many-Many* vs. *Few-Few*: −1.25 [−2.17 to −0.44]) and among late engagers (*Few-Many* vs. *Few-Few*: −1.18 [−2.20 to −0.08]), a finding also supported by additional finer-grained pattern analyses. Unlike lesson completion, the benefit of early worksheet completion did not persist through week 26.

Overall, our findings are in line with prior systematic reviews showing that over half of studies on digital CBT have reported a positive correlation between engagement and depressive symptom improvement, that is, greater engagement generally predicts better outcomes (Donkin et al., 2011). However, most previous studies did not adjust for post-baseline confounders, meaning that associations could reflect reverse causality (for example, participants increasing use because their symptoms improved). By applying a time-varying exposure approach, our study strengthens the evidence that higher engagement is indeed beneficial, independent of symptom trajectories, motivation shifts, or life events occurring during treatment.

Another advantage of this time-varying approach is the ability to capture dynamic patterns of engagement. By distinguishing minimal engagers (*Few-Few* pattern), early engagers (*Many-Few*), late engagers (*Few-Many*), and consistent high engagers (*Many-Many*), we were able to assess how changes in engagement influence outcomes over time. Although our dichotomized categorization may be a simplification, the four patterns were broadly consistent with recent studies that used machine-learning clustering methods to identify longitudinal engagement trajectories in digital mental health interventions (Aschbacher et al., 2023; Chien et al., 2020). Despite differences in engagement definition, our findings resonate with these studies in showing that even low engagers achieved some improvement, but various higher engagement patterns were generally linked to greater depression severity reduction.

Additionally, our study indicated a potential difference between lesson and worksheet engagement. For lesson completion, early engagers (*Many-Few*) achieved the largest PHQ-9 improvements, which persisted to 26 weeks. This aligns with the design of our smartphone app, where core BA skills were introduced in the initial sessions and, once learned, might continue to influence outcomes. In contrast, worksheets, which reflect active practice of BA skills, were associated with greater short-term improvements among those with later or consistently high engagement (*Few-Many* and *Many-Many*). One possible explanation is that the added benefit of worksheet engagement may depend on first acquiring the basic skills through lessons, making later engagement particularly influential. In the long term, however, continued engagement with worksheets was no longer associated with additional benefit, suggesting that once users have acquired the practical skills, they might not need to report their BA activities on the app, as they could perform them in their mind. Together, these findings may suggest that future digital CBT programs could promote early lesson engagement for rapid skill acquisition, while also supporting practice through homework or activities. In fact, digital platforms may offer unique opportunities to implement strategies that are difficult to achieve in traditional face-to-face therapy. For instance, automated encouragement emails or a chatbot could be used – particularly in the early stages to enhance adherence (Yasukawa et al., 2024). In addition, with growing interest in AI-based algorithms, lesson and worksheet delivery may in the future be dynamically tailored to participants’ individual engagement patterns (Furukawa et al., 2025a).

This study has several strengths. First, the g-formula approach allows us to account for post-baseline confounders, providing a more robust understanding of how engagement influences outcomes independent of symptom changes or motivation shifts. Second, our data come from a large-scale RCT of a smartphone CBT app in a general population sample with subthreshold depression in Japan. The trial collected rich longitudinal data, including depression severity, lesson and worksheet completion, motivation and satisfaction with the app use, and life events, all repeatedly measured with minimal missing data, enabling the application of this approach (Furukawa et al., 2023, 2025b). These features make the findings informative for guiding the design of future smartphone-based CBT programs and underscore their potential implications for both clinical practice and research.

Our study also has limitations. First, it is a post-hoc secondary analysis with an exploratory nature, so results should be interpreted with caution. Second, although the data come from an RCT, the analysis examines the association between engagement and outcomes in an observational framework; thus, unmeasured confounders could exist. Also, due to the limited sample size, we could not include additional time points or apply more complex models to investigate the interactions between lesson and worksheet completion, although the interplay between engagement and depression severity must arguably be more continually reciprocal. Nevertheless, our dataset included key post-baseline confounders such as motivation, expectations, and life events (Aziz et al., 2023; Boucher & Raiker, 2024). Third, engagement was operationalized by categorizing continuous completion counts into discrete patterns, which entails an inherent risk that alternative threshold definitions could yield different results. Although thresholds were chosen based on clinical interpretability, primarily with statistical feasibility considerations rather than data-driven selection, some degree of information loss and threshold dependence is unavoidable. Consequently, the observed associations should be interpreted as pattern-level, clinically oriented contrasts rather than exact dose–response relationships. Fourth, because the data were derived from a closely monitored RCT, the overall motivation and adherence were high, which may limit the generalizability to more heterogeneous engagement patterns in real-world settings (Fleming et al., 2018). However, the engagement patterns and their relationship with depression improvements in our study were broadly consistent with findings from real-world app use data (Aschbacher et al., 2023; Chien et al., 2020). Future studies may explore different engagement dimensions across diverse contexts for various CBT skill interventions based on larger samples.

## Conclusions

Our findings strengthen the evidence that, in smartphone-delivered BA intervention, greater engagement with lessons and worksheets is associated with larger reductions in depressive symptoms among individuals with subthreshold depression, independent of symptom changes, motivation shifts, or life events over time. Early engagement in both lessons and worksheets appears particularly important for better outcomes. While the benefits of lesson engagement extend to the long term, the benefits of worksheet engagement seem to diminish after the intervention. These findings may inform the design of future digital CBT programs by emphasizing strategies that promote early lesson engagement alongside simultaneous skill practice to optimize outcomes.

## Supporting information

10.1017/S0033291726104279.sm001 Luo et al. supplementary material Luo et al. supplementary material

## Data Availability

Deidentified individual participant data of the original RCT will be made available 24 months after the publication of the primary results on UMIN-ICDR, an individual case data repository managed by the Japanese University Hospital Medical Information Network (UMIN) Center (https://www.umin.ac.jp/icdr/index.html). Proposals with specific aims and an analysis plan should be directed to the corresponding author of the original trial, Professor Tosh A. Furukawa (furukawa@kuhp.kyoto-u.ac.jp).

## Supplementary material

The supplementary material for this article can be found at http://doi.org/10.1017/S0033291726104279.
