# Supplementary material for: Smartphone CBT engagement and depressive symptoms: secondary analysis of the RESiLIENT trial using a time-varying exposure approach
Source: Psychol Med. 2026 May 13;56:e145. doi: 10.1017/S0033291726104279 (PMC13200147; doi:10.1017/S0033291726104279)
Supplement: Luo et al. supplementary material [file S0033291726104279sup001.docx]

**Smartphone CBT Engagement and Depressive Symptoms: Secondary Analysis of the RESiLIENT Trial Using a Time-varying Exposure Approach**

Yan Luo, Kosuke Inoue, Aran Tajika, Rie Toyomoto, Masatsugu Sakata, Tatsuo Akechi,

Masaru Horikoshi, Hisashi Noma, Toshi A. Furukawa

**Supplementary Material**

[S1. Supplementary Methods 2](#_Toc220412303)

[S1.1 Summary of time-varying exposures. 2](#_Toc220412304)

[S1.2 Causal directed acyclic graph (DAG) for the association between the exposure and outcome. 3](#_Toc220412305)

[S2. Supplementary Results 4](#_Toc220412306)

[S2.1 Exposure 1: Lesson completion 4](#_Toc220412307)

[Figure S1. Distribution of total lessons completed. 4](#_Toc220412308)

[Table S1. Baseline characteristics of the four patterns for lesson completion over time. 4](#_Toc220412309)

[S2.2 Exposure 2: Worksheet completion 6](#_Toc220412310)

[Figure S2. Distribution of total worksheets completed. 6](#_Toc220412311)

[Table S2. Association between the total number of worksheets and change in PHQ-9 scores. 6](#_Toc220412312)

[Table S3. Baseline characteristics of the four patterns for worksheet completion over time. 6](#_Toc220412313)

[Table S4. Results for time-varying worksheet completion (Sensitivity analyses). 7](#_Toc220412314)

[Figure S3. Association between worksheet completion patterns and changes in PHQ-9 scores at week 6 (Sensitivity analyses). 9](#_Toc220412315)

# S1. Supplementary Methods

## S1.1 Summary of time-varying exposures.

|  | N during week 0-3 | N during week 3-6 | N during week 6-26 |  |  |
| --- | --- | --- | --- | --- | --- |
| Exposure 1: Lessons | | | |  |  |
| *Few-Few* pattern  [Minimal engagement] | ≤ 3 | ≤ 2 | - |  |  |
| *Few-Many* pattern  [Late engagement] | ≤ 3 | ≥ 3 | - |  |  |
| *Many-Few* pattern  [Early engagement] | ≥ 4 | ≤ 2 | - |  |  |
| *Many-Many* pattern  [High engagement] | ≥ 4 | ≥ 3 | - |  |  |
| Exposure 2: Number of worksheets | | | |  |  |
| (1)-1 Before week 6: Main analysis | | | |  |  |
| *Few-Few* pattern  [Minimal engagement] | ≤ 10 | ≤ 5 | - |  |  |
| *Few-Many* pattern  [Late engagement] | ≤ 10 | ≥ 6 | - |  |  |
| *Many-Few* pattern  [Early engagement] | ≥ 11 | ≤ 5 | - |  |  |
| *Many-Many* pattern  [High engagement] | ≥ 11 | ≥ 6 | - |  |  |
| (1)-2 Before week 6: Sensitivity analysis (a) | | | |  |  |
| *Few-Few* pattern | ≤ 10 | ≤ 5 | - |  |  |
| *Few-Moderate* pattern | ≤ 10 | 6-10 | - |  |  |
| *Few-Many* pattern | ≤ 10 | ≥ 11 | - |  |  |
| *Moderate-Few* pattern | 11-20 | ≤ 5 | - |  |  |
| *Moderate-Moderate* pattern | 11-20 | 6-10 | - |  |  |
| *Moderate-Many* pattern | 11-20 | ≥ 11 | - |  |  |
| *Many-Few* pattern | ≥ 21 | ≤ 5 | - |  |  |
| *Many-Moderate* pattern | ≥ 21 | 6-10 | - |  |  |
| *Many-Many* pattern | ≥ 21 | ≥ 11 |  |  |  |
| (1)-3 Before week 6: Sensitivity analysis (b) | | | |  |  |
| *Few-Few* pattern | ≤ 8 | ≤ 4 | - |  |  |
| *Few-Moderate* pattern | ≤ 8 | 5-10 | - |  |  |
| *Few-Many* pattern | ≤ 8 | ≥ 11 | - |  |  |
| *Moderate-Few* pattern | 9-17 | ≤ 4 | - |  |  |
| *Moderate-Moderate* pattern | 9-17 | 5-10 | - |  |  |
| *Moderate-Many* pattern | 9-17 | ≥ 11 | - |  |  |
| *Many-Few* pattern | ≥ 18 | ≤ 4 | - |  |  |
| *Many-Moderate* pattern | ≥ 18 | 5-10 | - |  |  |
| *Many-Many* pattern | ≥ 18 | ≥ 11 |  |  |  |
| (2) Before week 26: | | | |  | ≥ 6 |
| *Few-Few-Few* pattern | ≤ 10 | ≤ 5 | ≤ 3 |  |  |
| *Few-Few-Many* pattern | ≤ 10 | ≤ 5 | ≥ 4 |  |  |
| *Few-Many-Few* pattern | ≤ 10 | ≥ 6 | ≤ 3 |  |  |
| *Few-Many-Many* pattern | ≤ 10 | ≥ 6 | ≥ 4 |  |  |
| *Many-Few-Few* pattern | ≥ 11 | ≤ 5 | ≤ 3 |  |  |
| *Many-Few-Many* pattern | ≥ 11 | ≤ 5 | ≥ 4 |  |  |
| *Many-Many-Few* pattern | ≥ 11 | ≥ 6 | ≤ 3 |  |  |
| *Many-Many-Many* pattern | ≥ 11 | ≥ 6 | ≥ 4 |  |  |

## S1.2 Causal directed acyclic graph (DAG) for the association between the exposure and outcome.

1. Fixed exposure approach (baseline confounders)

1. Time-varying exposure approach

*Baseline confounder set L0: refer to (A).

# S2. Supplementary Results

## S2.1 Exposure 1: Lesson completion

### Figure S1. Distribution of total lessons completed.


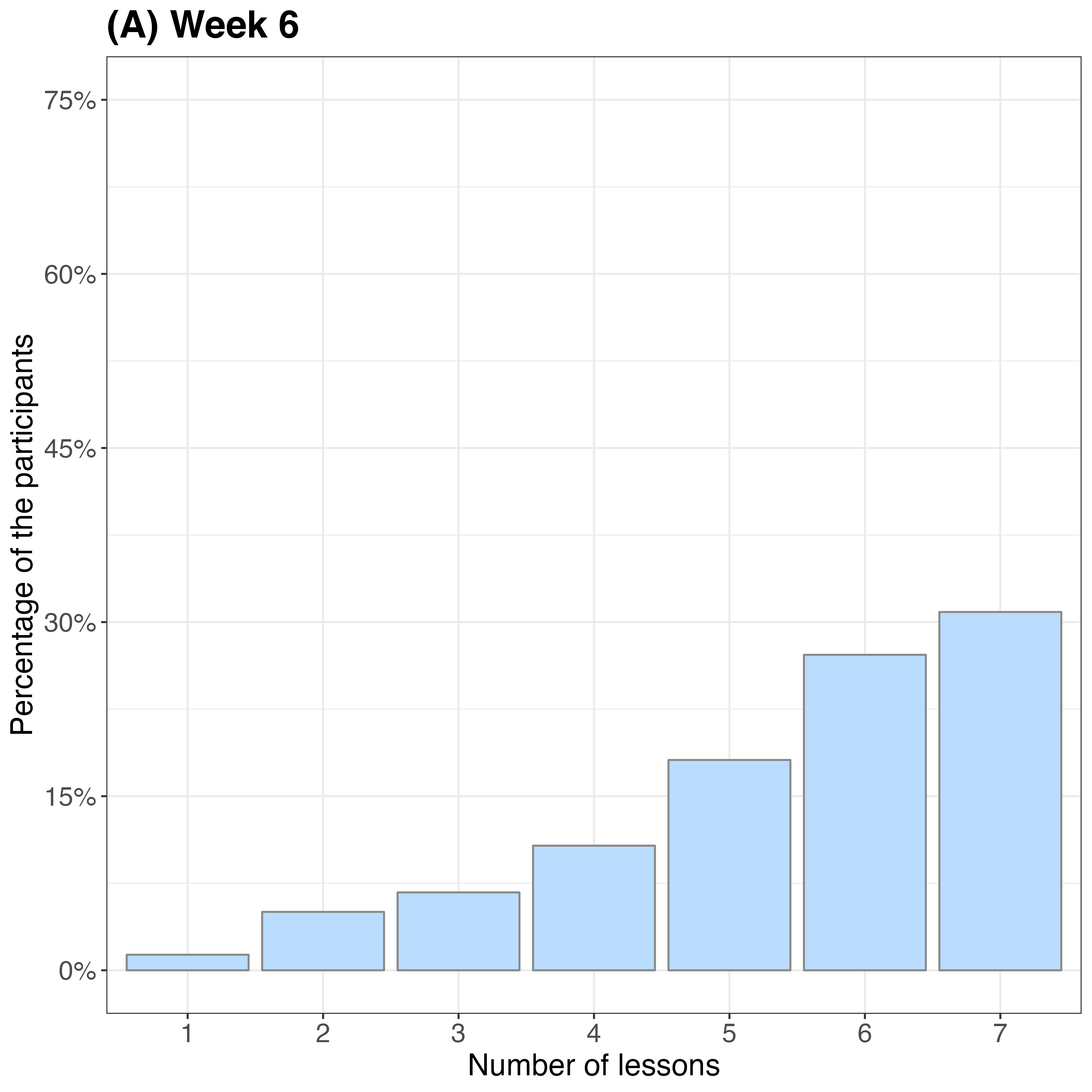

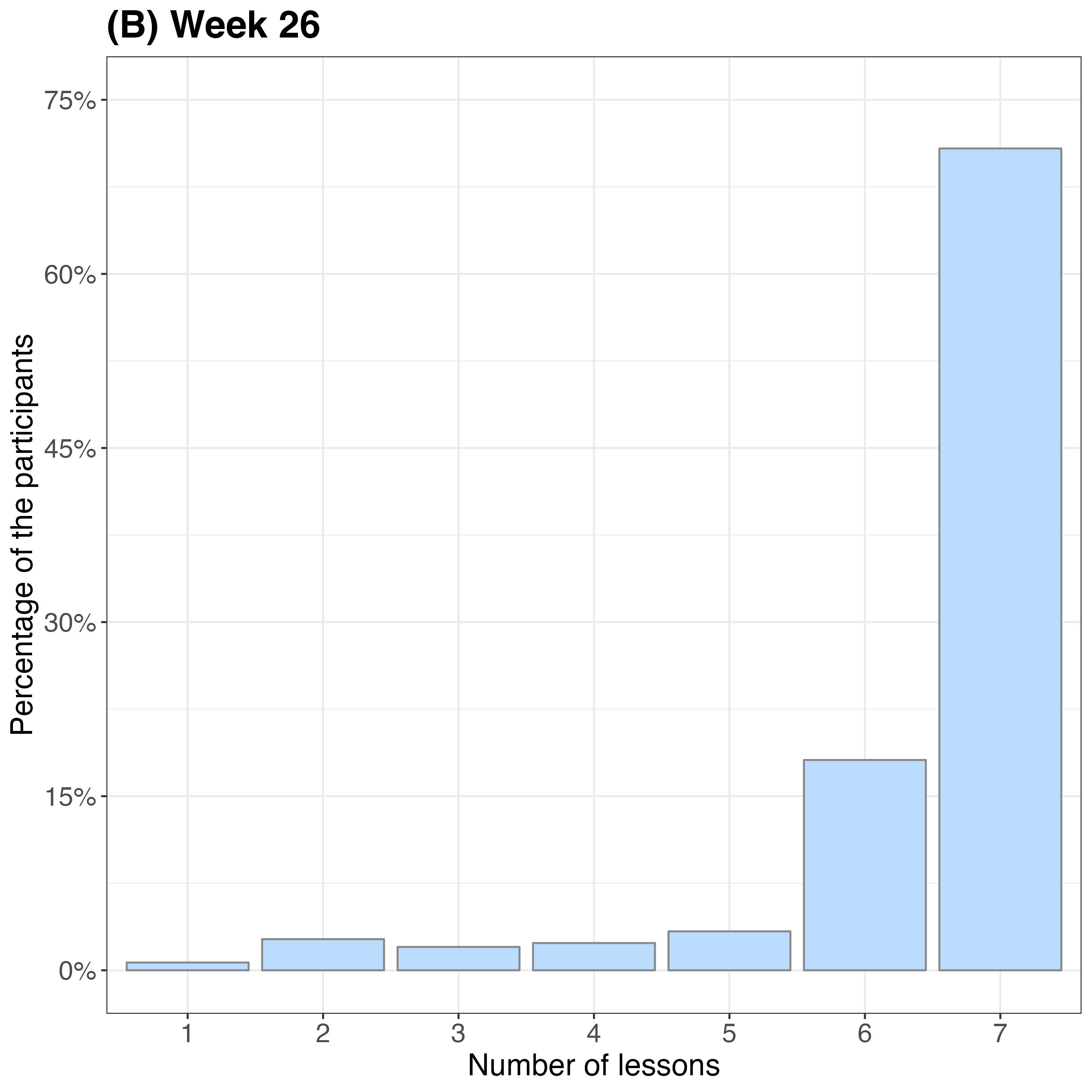


### Table S1. Baseline characteristics of the four patterns for lesson completion over time.

|  | Few-Few | Few-Many | Many-Few | Many-Many |
| --- | --- | --- | --- | --- |
| N | 85 | 61 | 85 | 67 |
| Age, mean (SD) | 44.2 (11.1) | 45.1 (11.0) | 44.9 (10.0) | 46.0 (11.0) |
| Sex, n (%) |  |  |  |  |
| Female | 37 (43.5) | 23 (37.7) | 46 (54.1) | 44 (65.7) |
| Male | 48 (56.5) | 37 (60.7) | 39 (45.9) | 22 (32.8) |
| Other | 0 (0) | 1 (1.6) | 0 (0) | 1 (1.5) |
| Number of cohabitants including self, mean (SD) | 2.59 (1.24) | 2.77 (1.38) | 2.88 (1.29) | 2.75 (1.31) |
| Education, University or higher, n (%) | 55 (64.7) | 47 (77.0) | 62 (72.9) | 45 (67.2) |
| Employment, Employed, n (%) | 80 (94.1) | 58 (95.1) | 78 (91.8) | 56 (83.6) |
| Big-5 personality traits, mean (SD) |  |  |  |  |
| Neuroticism | 3.66 (0.77) | 3.51 (0.83) | 3.57 (0.77) | 3.66 (0.78) |
| Extraversion | 2.90 (0.84) | 2.97 (0.99) | 2.93 (0.94) | 3.04 (0.98) |
| Openness | 3.22 (0.74) | 3.23 (0.76) | 3.11 (0.80) | 3.11 (0.77) |
| Conscientiousness | 2.92 (0.72) | 3.01 (0.85) | 3.08 (0.81) | 3.06 (0.78) |
| Agreeableness | 3.46 (0.83) | 3.09 (0.94) | 3.03 (0.78) | 3.14 (0.89) |
| Assessment of Signal Cases, mean (SD) |  |  |  |  |
| Social support | 1.37 (0.78) | 1.11 (0.66) | 1.39 (0.71) | 1.41 (0.77) |
| Life difficulties | 0.90 (0.60) | 0.97 (0.57) | 0.93 (0.57) | 0.95 (0.61) |
| Motivation | 2.22 (0.37) | 2.12 (0.39) | 2.23 (0.37) | 2.25 (0.35) |
| PHQ-9, mean (SD) | 7.65 (2.71) | 8.15 (2.86) | 8.31 (2.36) | 7.52 (2.21) |
| Past or current treatments for mental health, n (%) | 32 (37.6) | 15 (24.6) | 28 (32.9) | 23 (34.3) |

Abbreviations: PHQ-9, Patient Health Questionnaire-9; SD, standard deviation.

## S2.2 Exposure 2: Worksheet completion

### Figure S2. Distribution of total worksheets completed.


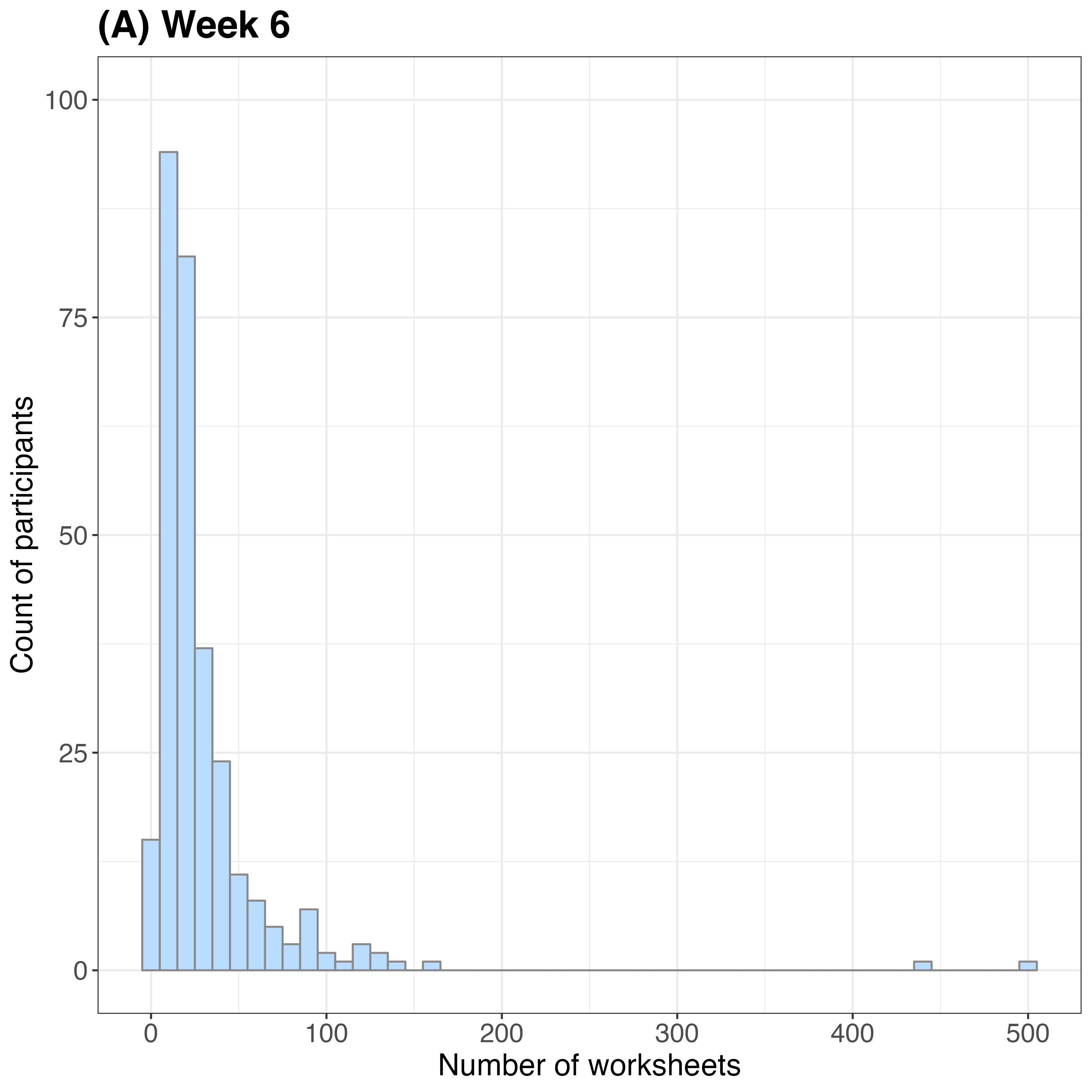

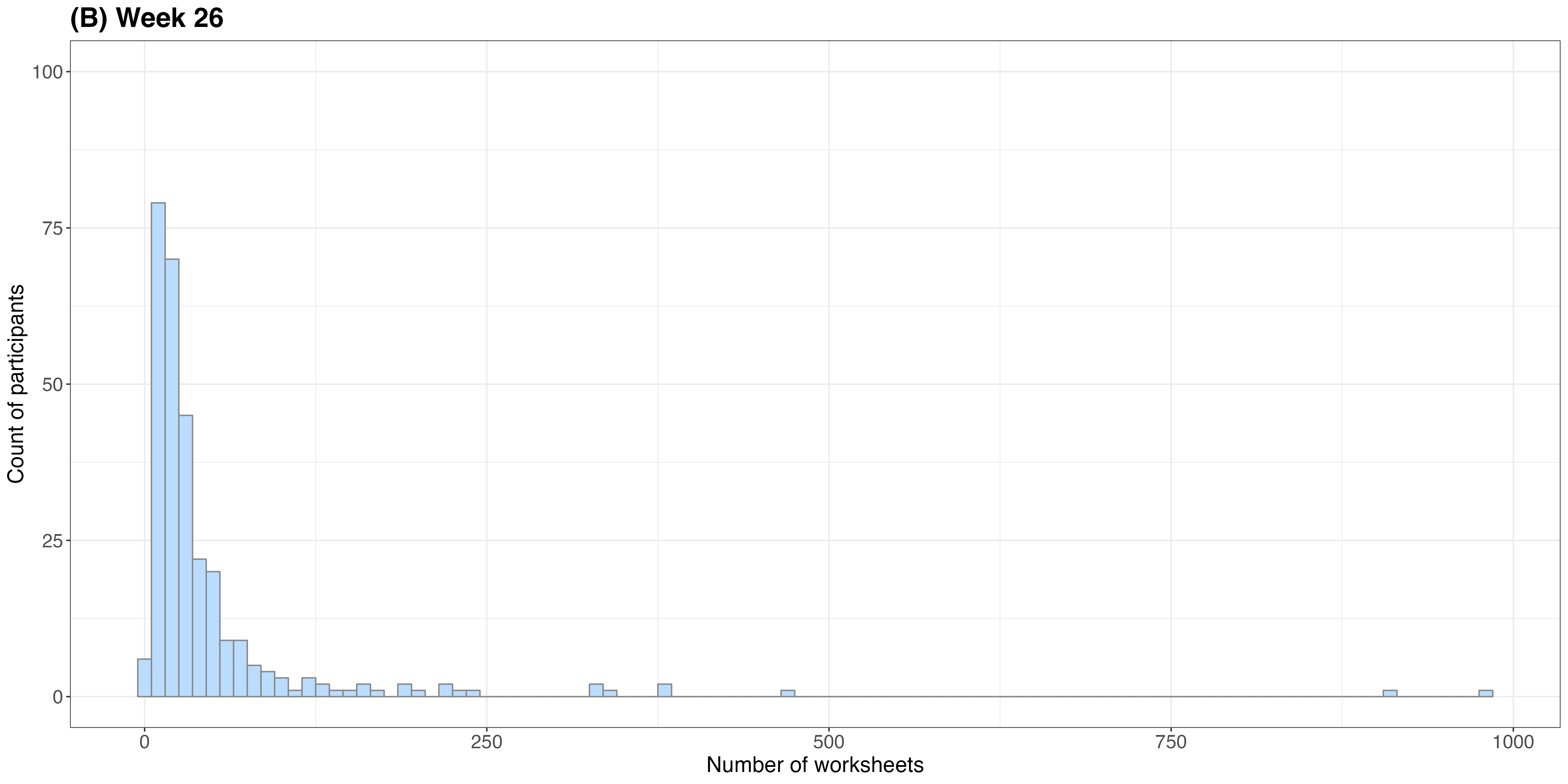


### Table S2. Association between the total number of worksheets and change in PHQ-9 scores.

| Week 6 | | | Week 26 | | |
| --- | --- | --- | --- | --- | --- |
| Total number of worksheets by week 6 | **Association with PHQ-9 change** | **P value** | **Total number of worksheets by week 26** | **Association with PHQ-9 change** | **P value** |
| 0-10 | Reference | - | **0-10** | Reference | - |
| 11-15 | -0.58 (-1.66, 0.50) | 0.29 | **11-15** | -0.28 (-1.89, 1.33) | 0.73 |
| 16-20 | -0.83 (-1.90, 0.23) | 0.12 | **16-20** | -0.71 (-2.24, 0.81) | 0.36 |
| 21-30 | -1.41 (-2.53, -0.29)* | 0.01 | **21-30** | -0.05 (-1.69, 1.58) | 0.95 |
| 31-40 | -1.04 (-2.30, 0.20) | 0.10 | **31-40** | -1.22 (-2.99, 0.56) | 0.18 |
| ≥ 41 | -1.07 (-2.17, 0.02) | 0.06 | **≥ 41** | 0.32 (-1.14, 1.78) | 0.67 |

Abbreviations: PHQ-9, Patient Health Questionnaire-9.

### Table S3. Baseline characteristics of the four patterns for worksheet completion over time.

|  | Few-Few | Few-Many | Many-Few | Many-Many |
| --- | --- | --- | --- | --- |
| N | 75 | 55 | 40 | 128 |
| Age, mean (SD) | 43.75 (11.15) | 43.49 (11.05) | 39.80 (10.97) | 47.96 (9.37) |
| Sex, n (%) |  |  |  |  |
| Female | 30 (40.0) | 26 (47.3) | 20 (50.0) | 74 (57.8) |
| Male | 45 (60.0) | 28 (50.9) | 20 (50.0) | 53 (41.4) |
| Other | 0 (0) | 1 (1.8) | 0 (0) | 1 (0.8) |
| Number of cohabitants including self, mean (SD) | 2.79 (1.28) | 2.58 (1.36) | 2.70 (1.34) | 2.80 (1.27) |
| Education, University or higher, n (%) | 52 (69.3) | 35 (63.6) | 31 (77.5) | 91 (71.1) |
| Employment, Employed, n (%) | 68 (90.7) | 52 (94.5) | 37 (92.5) | 115 (89.8) |
| Big-5 personality traits, mean (SD) |  |  |  |  |
| Neuroticism | 3.57 (0.85) | 3.59 (0.79) | 3.68 (0.67) | 3.61 (0.78) |
| Extraversion | 2.73 (0.83) | 3.07 (0.98) | 3.07 (0.92) | 3.00 (0.96) |
| Openness | 3.06 (0.71) | 3.13 (0.79) | 3.00 (0.73) | 3.29 (0.79) |
| Conscientiousness | 3.07 (0.76) | 2.84 (0.86) | 2.72 (0.66) | 3.15 (0.77) |
| Agreeableness | 3.23 (0.80) | 3.24 (0.93) | 3.34 (0.96) | 3.10 (0.84) |
| Assessment of Signal Cases, mean (SD) |  |  |  |  |
| Social support | 1.35 (0.76) | 1.22 (0.75) | 1.28 (0.69) | 1.39 (0.74) |
| Life difficulties | 0.83 (0.61) | 0.90 (0.55) | 1.02 (0.54) | 0.99 (0.60) |
| Motivation | 2.17 (0.37) | 2.21 (0.36) | 2.20 (0.38) | 2.23 (0.37) |
| PHQ-9, mean (SD) | 7.49 (2.37) | 8.00 (2.87) | 8.40 (2.73) | 7.96 (2.43) |
| Past or current treatments for mental health, n (%) | 28 (37.3) | 14 (25.5) | 16 (40.0) | 40 (31.2) |

Abbreviations: PHQ-9, Patient Health Questionnaire-9; SD, standard deviation.

### Table S4. Results for time-varying worksheet completion (Sensitivity analyses).

| Sensitivity analysis (a) | | | | | | |
| --- | --- | --- | --- | --- | --- | --- |
| Pattern | **N** | **Number of worksheets in weeks 0-3** | **Number of worksheets in weeks 3-6** | **Total number of worksheets**, median (Q1, Q3) | **Estimated PHQ-9 change from baseline** | **Estimated mean difference** |
| *Few-Few* | 75 | ≤ 10 | ≤ 5 | 9 (6.5, 11) | -2.76  (-3.39 to -2.16) | Reference |
| *Few-Moderate* | 41 | ≤ 10 | 6-10 | 15 (11, 16) | -3.39  (-3.98 to -2.81) | -0.64  (-1.30 to 0.13) |
| *Few-Many* | 14 | ≤ 10 | ≥ 11 | 21.5 (19, 26.5) | -4.03  (-5.15 to -2.79) | -1.27  (-2.60 to 0.26) |
| *Moderate-Few* | 33 | 11-20 | ≤ 5 | 17 (14, 18) | -3.35  (-4.12 to -2.71) | -0.60  (-1.35 to 0.13) |
| *Moderate-Moderate* | 36 | 11-20 | 6-10 | 22.5 (20, 25) | -3.68  (-4.14 to -3.29) | -0.93  (-1.66 to -0.28)* |
| *Moderate-Many* | 20 | 11-20 | ≥ 11 | 33.5 (29.75, 38.25) | -4.02  (-4.67 to -3.33) | -1.26  (-2.26 to -0.24)* |
| *Many-Few* | 7 | ≥ 21 | ≤ 5 | 32 (27, 34.5) | -3.95  (-5.28 to -2.72) | -1.19  (-2.69 to 0.26) |
| *Many-Moderate* | 18 | ≥ 21 | 6-10 | 34 (32, 39.75) | -3.98  (-4.75 to -3.28) | -1.22  (-2.26 to -0.26)* |
| *Many-Many* | 54 | ≥ 21 | ≥ 11 | 64.5 (45.5, 94) | -4.01  (-4.95 to -3.16) | -1.25  (-2.37 to -0.17)* |
| Sensitivity analysis (b) | | | | | | |
| Pattern | **N** | **Number of worksheets in weeks 0-3** | **Number of worksheets in weeks 3-6** | **Total number of worksheets**, median (Q1, Q3) | **Estimated PHQ-9 change from baseline** | **Estimated mean difference** |
| *Few-Few* | 58 | ≤ 8 | ≤ 4 | 8 (6, 9) | -2.51  (-3.17 to -1.79) | Reference |
| *Few-Moderate* | 32 | ≤ 8 | 5-10 | 13 (10, 15) | -3.31  (-3.98 to -2.69) | -0.80  (-1.57 to -0.07)* |
| *Few-Many* | 10 | ≤ 8 | ≥ 11 | 20 (19, 22) | -4.11  (-5.36 to -2.93) | -1.60  (-3.14 to -0.14)* |
| *Moderate-Few* | 36 | 9-17 | ≤ 4 | 14 (12.75, 17) | -3.21  (-3.92 to -2.55) | -0.70  (-1.48 to 0.02) |
| *Moderate-Moderate* | 49 | 9-17 | 5-10 | 19 (17, 22) | -3.67  (-4.10 to -3.27) | -1.17  (-1.99 to -0.42)* |
| *Moderate-Many* | 18 | 9-17 | ≥ 11 | 31.5 (27, 35.75) | -4.14  (-4.82 to -3.46) | -1.63  (-2.78 to -0.58)* |
| *Many-Few* | 6 | ≥ 18 | ≤ 4 | 27 (22.5, 31.5) | -3.92  (-5.18 to -2.66) | -1.41  (-2.95 to 0.04) |
| *Many-Moderate* | 29 | ≥ 18 | 5-10 | 32 (29, 36) | -4.04  (-4.76 to -3.37) | -1.53  (-2.62 to -0.53)* |
| *Many-Many* | 60 | ≥ 18 | ≥ 11 | 62 (43.25, 91.75) | -4.17  (-4.99 to -3.35) | -1.66  (-2.80 to -0.55)* |

Abbreviations: PHQ-9, Patient Health Questionnaire-9.

### Figure S3. Association between worksheet completion patterns and changes in PHQ-9 scores at week 6 (Sensitivity analyses).


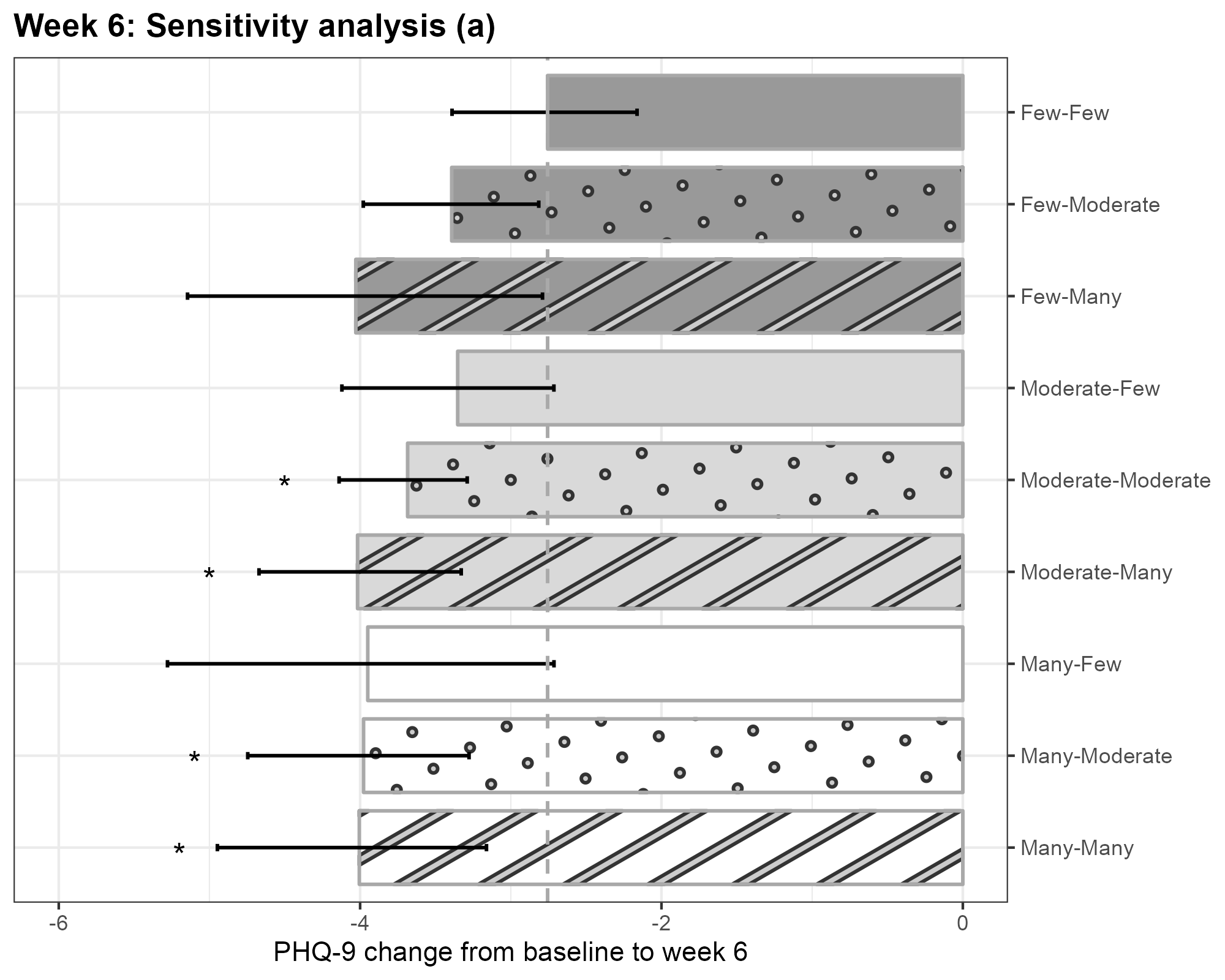


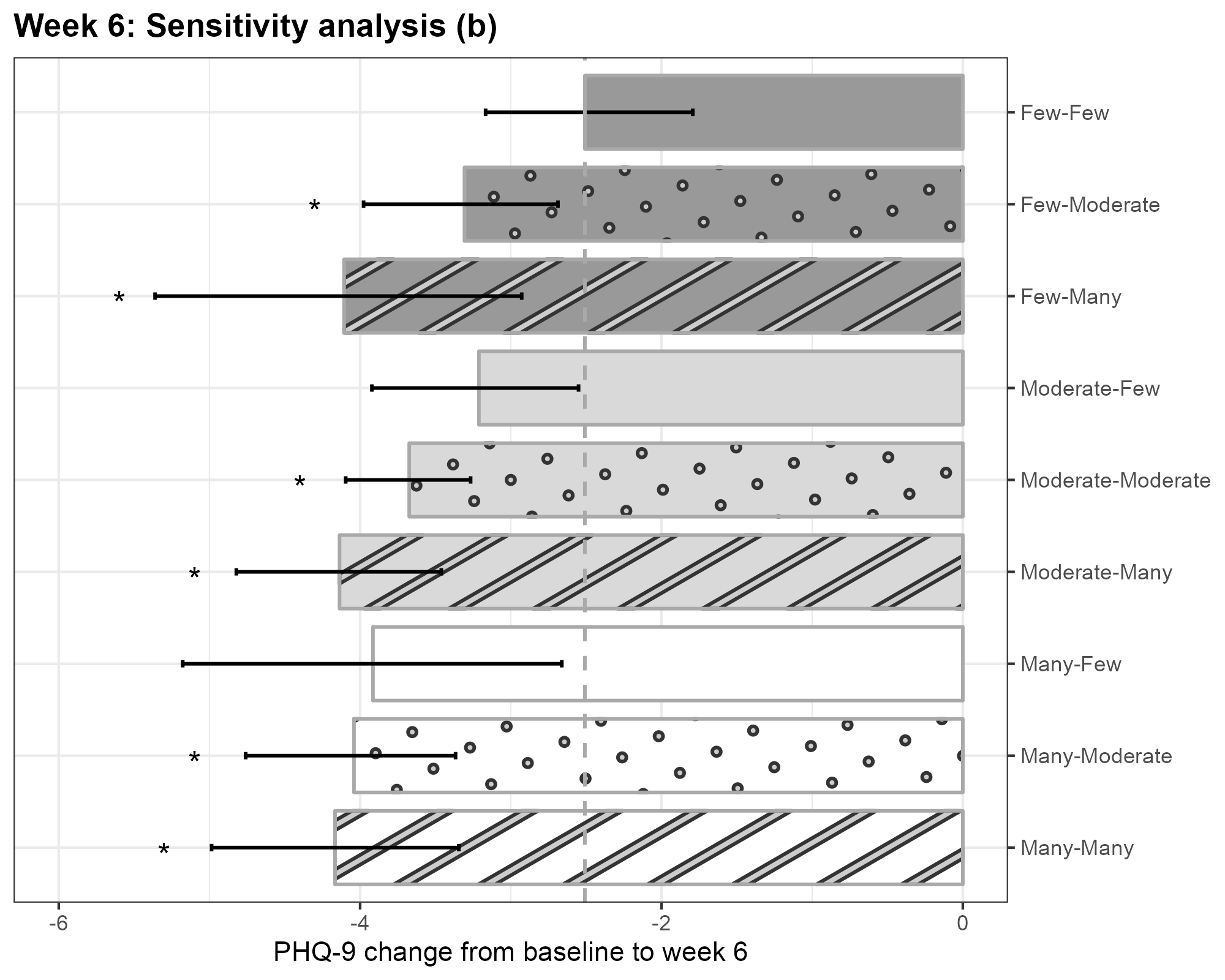


The bars marked with * indicate that the bootstrap 95% confidence intervals for the mean difference between those groups and the *Few–Few* pattern do not include the null value, suggesting greater improvements compared with the minimal engagement group. Abbreviations: PHQ-9, Patient Health Questionnaire-9.
